# Influence of Selenium Biofortification on the Growth and Bioactive Metabolites of *Ganoderma lucidum*

**DOI:** 10.3390/foods10081860

**Published:** 2021-08-12

**Authors:** Mengmeng Xu, Song Zhu, Lingling Wang, Zhiyi Wei, Liting Zhao, Guiyang Shi, Zhongyang Ding

**Affiliations:** 1 Key Laboratory of Carbohydrate Chemistry and Biotechnology, Ministry of Education & School of Biotechnology, Jiangnan University, Wuxi 214122, China; xmm900801@163.com (M.X.); wanglingling@jiangnan.edu.cn (L.W.); weizhiyi0619@163.com (Z.W.); zhaoliting1num@163.com (L.Z.); gyshi@jiangnan.edu.cn (G.S.); 2 National Engineering Laboratory for Cereal Fermentation Technology, Jiangnan University, Wuxi 214122, China; 3 State Key Laboratory of Food Science and Technology, School of Food Science and Technology, Jiangnan University, Wuxi 214122, China; zhusong@jiangnan.edu.cn; 4 Jiangsu Provincial Research Center for Bioactive Product Processing Technology, Jiangnan University, Wuxi 214122, China

**Keywords:** selenite, volatile selenium compound, selenoamino acid, polysaccharide, edible and medicinal mushroom, liquid culture, morphology, ultra-structure

## Abstract

Selenium biofortification of edible and medicinal mushrooms is an effective way to produce selenium-enriched food supplements. *Ganoderma lucidum* is the typical one with excellent biological activity. This study investigated *G. lucidum* growth and bioactive metabolites alterations during liquid culture with different concentrations of selenite. Low selenium levels did not affect growth and mycelia morphology, whereas high selenium levels negatively influenced growth, dramatically decreased biomass, caused nucleic acid and protein leakage, damaged cell walls and membranes, and resulted in indicators such as degraded cells, a red color, and an unpleasant odor. Through headspace-solid phase microextraction-gas chromatography-mass spectrometry (HS-SPME-GC-MS) analysis, ten volatile Se compounds were identified in *G. lucidum* with 200 ppm selenite, which led to an odor change, whereas only three with 50 ppm selenite. SeMet was the major selenoamino acid in the 50 ppm selenite group by high-performance liquid chromatography-inductively coupled plasma mass spectrometry (HPLC-ICP-MS), but more MeSeCys was produced with 200 ppm selenite. Polysaccharide yields were promoted and inhibited with 50 and 200 ppm selenite, respectively. These results provide comprehensive insights into the effects of selenite on *G. lucidum* in liquid culture and are beneficial for functional selenium-enriched mushroom production and improving nutritive values.

## 1. Introduction

Selenium (Se), an essential micronutrient for humans, participates in a series of cellular metabolic processes and possesses vital biological functions, specifically with potent antioxidant, anticancer, anti-inflammatory, and antiviral properties, among others [1,2]. Plants and microorganisms have been developed as major Se biofortification sources and are considered as dominant functional foods or food ingredients of daily Se supplementation. Se-enriched arable crops, vegetable crops, and Se-yeast and Se-algae are presently recommended as the primary sources of dietary Se for humans [3,4,5].

Edible and medicinal mushrooms are widely consumed as favorable food supplements because they are rich in polysaccharides, proteins, and other nutrients, thereby having multiple nutritive values [6]. The production of functional mushrooms by Se biofortification is an effective way to improve the demand for Se in humans due to their strong capacity to uptake and accumulate this element [7]. Mushrooms can transform inorganic Se into organic Se by incorporating it into active macromolecules during metabolism [8]. The bioaccumulation of Se and their bioactive metabolites is associated with elevated nutraceutical properties in many mushroom species, such as antimicrobial and antioxidant activities in *Coriolus versicolor* [9], antioxidant activity and flavor properties in *Pleurotus eryngii* [10], and immunomodulatory effects in *Grifola frondosa* [11].

Current research on Se-enriched mushrooms has mainly centered on fruiting body cultivation by adding inorganic Se compounds to solid substrates, further investigating Se metabolite structural characterizations and the potential bioactivities. Se enrichment ability varies among mushroom species, culture conditions, Se sources, and doses. *C. versicolor* biomass decreases much more with Na_2_SeO_3_ than with selenourea addition, and selenourea has less impact and lower toxicity on mycelium growth than Na_2_SeO_3_ [9]. However, Se concentrations and forms significantly affect the *Hericium erinaceus* mycelium and fruiting body growth, and both respond better to inorganic forms [12]. The growth rate of *Flammulina velutipes* is suppressed at lower selenite concentrations in solid and static cultivation as compared to that with shaking cultivation [13].

In comparison to solid cultivation, liquid culture has a shorter production cycle and higher production benefit [14]. It is a promising application direction to obtain large mycelium yields or bioactive metabolites via liquid culture technology, which could be developed into various functional foods in the food industry. However, few studies have explored the influence of Se on mushroom mycelia during liquid culture.

*Ganoderma lucidum*, a traditional valuable source of nutrients for centuries, has a high amount of polysaccharide and is considered functional due to its anticancer, antioxidant, and anti-inflammatory activities [15,16]. This study used *G. lucidum* as the research object and compared the growth and bioactive metabolites alterations in mycelia cultured with different concentrations of selenite, providing evidence in terms of pellet morphology, cell structure components, and odor change. The total Se content, selenoamino acids, and polysaccharide were also analyzed to define variations in the major bioactive metabolites. This study provides comprehensive insights into the influence of Se on *G. lucidum* mycelia during the liquid culture process, and is useful for the production of Se-enriched mushrooms with high nutritive values.

## 2. Materials and Methods

### 2.1. Reagents and Standards

Solvents and chemicals of reagent grade were used. All aqueous solutions were prepared by deionized water (18.2 MΩ cm^−1^), which was obtained using a Milli-Q system (Bedford, MA, USA). Sodium selenite [Se (IV)] was obtained from the China National Pharmaceutical Industry Corporation, Ltd. SeCys_2_ (GBW10087), MeSeCys (GBW10088), and SeMet (GBW10034) were purchased from the Center for Standard Reference (PRC).

### 2.2. Strain and Culture Conditions

*Ganoderma lucidum* (CGMCC 5.26) was purchased from the China General Microbiological Culture Collection Center (CGMCC) and preserved on potato dextrose agar slants at 4 °C. The seed and liquid culture medium was composed of glucose (20 g/L), yeast nitrogen base without amino acids (5 g/L), tryptone (5 g/L), KH_2_PO_4_ (4.5 g/L), MgSO_4_·7H_2_O (2 g/L), and 0–500 ppm Na_2_SeO_3_ at initial pH. Liquid culture was performed in a 500 mL flask with 150 mL medium and kept at 30 °C at 150 rpm on a rotary shaker.

### 2.3. Determination of Biomass, Residual Sugar, Polysaccharide Content

Mycelia were harvested by centrifugation at 10,000 rpm for 10 min. The culture broth volume was restored to its initial value by adding distilled water before centrifugation. Washing the precipitate three times with distilled water and then lyophilizing. Dry cell weight was determined using the gravimetric method [17]. The residual sugar content in the medium was measured using the 3,5-dinitrosalicylic acid method. Total polysaccharide content was calculated as the sum of intracellular and extracellular polysaccharide. Intracellular polysaccharide was extracted in hot water, and extracellular polysaccharide was obtained by ethanol precipitation, and their concentrations were determined using the phenol-sulfuric acid method, as described by Ma et al. (2019) [17].

### 2.4. Ultra-Structural Analysis

The transmission electron microscopy (TEM) method is commonly used to observe the ultrastructure of *G. lucidum* as described by Ma et al. (2019) [17]. Briefly, (1) fixing the cultured *G. lucidum* mycelia in 5% glutaraldehyde (0.1 M phosphate buffer, pH 7.2) and rinsing them with phosphate buffer (0.1 M); (2) fixing the cells again with 1% osmium acid (0.1 M phosphate buffer, pH 7.2) and rinsing them with phosphate buffer (0.1 M); (3) dehydrating the cell samples and embedding them in Epon 812 resin; (4) finally, staining the cell sections with saturated uranyl acetate and aqueous lead citrate solutions, and then observing the ultrastructure under a HITACHI H-7650.

### 2.5. Quantification of Nucleic Acid, Protein, Chitin, β-1,3-Glucan, and Ergosterol

Briefly, 1 mL of the supernatant obtained by centrifugation was tested for nucleic acid and protein leakage after an appropriate dilution by measuring the absorbance at 260 nm and 280 nm. β-1,3-glucan and chitin quantification was performed as described by Ma et al. (2019) [17] and Miyazawa et al. (2018) [18] with some modifications. The mycelium sample (40 mg) was suspended in 2 mL of 1 M NaOH solution, mixed, and incubated at 52 °C for 30 min. A total of 0.5 mL supernatant was collected in a new tube, and 1.85 mL of an aniline blue mix was added for incubation at 52 °C for 30 min. Fluorescence was detected using a fluorescence spectrophotometer (HITACHI F-7000) with 405 nm excitation and 460 nm emission. β-1,3-glucan content was expressed as the relative fluorescence unit percentage per milligram of mycelium.

To sample 30 mg mycelium, 3 mL of a saturated KOH solution was added and heated at 130 °C for 1 h. The sample was cooled to room temperature, thereafter, 8 mL chilled 75% (*v*/*v*) ethanol was added, and the sample was shaken well and then incubated in an ice bath for 5 min. Next, 0.9 mL of 13.3% (*w*/*v*) celtite545 was added, then the mixture was shaken for 5 min and then centrifuged at 4 °C at 5000 rpm for 5 min. The precipitate was washed with 10 mL of chilled 40% (*v*/*v*) ethanol and 10 mL of chilled distilled water. The sample was prepared by centrifugation to remove the solution; 20 mL distilled water was then added, and 0.5 mL of the mixture was taken as the sample.

A standard solution (10 mg/mL glucosamine) and a blank control (distilled water) were prepared. 0.5 mL of water, 5% (*w*/*v*) NaNO_2_, and 5% (*w*/*v*) KHSO_4_ were added to the sample (0.5 mL) and then gently mixed for 15 min. The solution was then centrifuged at 10,000 rpm for 5 min at 4 °C to remove the precipitate. Then, 0.5 mL of 12.5% (*w*/*v*) NH_4_ sulfamate was added to a 0.5 mL sample, which was mixed vigorously for 5 min, followed by the addition of 0.5 mL of 0.5% (*w*/*v*) 3-methyl benzothiazolinone-2- hydrazone solution. The samples were then mixed and boiled for 3 min. After cooling to room temperature, 0.5 mL of 0.83% (*w*/*v*) FeCl_3_·6H_2_O was added. Keeping the sample at room temperature for 30 min. The absorbance was measured with a spectrophotometer at room temperature for 650 nm. The glucosamine content decomposed by chitin can be calculated according to the formula:

$$X=\frac{A-A_{0}}{A_{1}-A_{0}}*10$$

where X is the glucosamine concentration (mg/mL), *A*_1_ is the standard absorbance value, *A*_0_ is the absorbance of distilled water, A is the absorbance of the sample, and 10 is the glucosamine concentration (mg/mL).

Ergosterol concentration was determined according to the method described by Wang et al. (2020) [19] and Yuan et al. (2006) [20] with some modifications. Ultrasonic extraction of lyophilized *G. lucidum* mycelia powder with methanol for 2 h. The supernatant was evaporated to dryness under a N_2_ atmosphere. The residue was saponified with a 10% (*w*/*v*) KOH–75% (*v*/*v*) ethanol solution at 50 °C for 2 h. The mixture was subjected to hexane extraction three times, and the hexane layer was collected and evaporated to dryness under N_2_. The residue was dissolved in methanol and subjected to high-pressure liquid chromatography (HPLC) on an Agilent 1260 (VWD/RID) apparatus equipped with a Symmetry C18 5.0 column (4.6 × 250 mm; Waters, Milford, MA, USA). The mobile phase used the solvent of 100% methanol, and the procedure was conducted as isocratic elution with a 1.0 mL/min constant flow rate. Ergosterol content was determined and monitored at a 282 nm UV wavelength. Identification and quantification were performed according to retention time, UV spectra, and ergosterol standard (Sigma Aldrich, St. Louis, MO, USA). The equation of the calibration curve was performed by plotting the peak areas (Y) against the concentrations (X) of ergosterol. The limit of detection (LOD) and the limit of quantification (LOQ) were measured with signal-to-noise (S/N) of 3:1 and 10:1, respectively. The calibration curve: Y = 34.427X + 40.669, R^2^ = 0.9981. The LOD and the LOQ with a 10 µL injection were 0.06 µg/mL and 0.10 µg/mL, respectively.

### 2.6. Determination of Total Se Content and Selenoamino Acid Analysis

Samples (50 mg) were digested with 3 mL of nitric acid (for trace metal analysis, CNW Technology) in a microwave-assisted digestion system (MARS6, CEM Corp., Matthews, NC, USA) at 250 °C for 20 min. The digests were brought to 100 mL volume in polypropylene flasks with deionized water. The total Se concentration was determined using an inductively coupled plasma mass spectrometer (ICP-MS; ICAP TQ, Thermo Fisher Scientific Inc. Germering, Germany). Selenium was measured using an M-TQ-O2 with oxygen at a flow rate of 3.5 mL min^−1^ at *m*/*z* 80. Terbium was used as an internal standard. Blanks and a certified reference material (GSB-29, pork liver, Center for Standard Reference, PRC) were included in each batch of samples for quality control and assurance. The GSB-29 recovery varied from 89% to 111%. Three biological replicates were performed for all samples.

Selenoamino acid analysis was performed as described previously [7]. Briefly, 100 mg of powdered freeze-dried mycelia samples was mixed with 15 mg of protease E (Sigma Chemical Co., St. Louis, MO, USA) and 7 mL 30 mM tris-HCl (pH 7.5) and incubated for 8 h at 40 °C with shaking. The samples were centrifuged at 12,000 rpm at 4 °C for 10 min. The supernatant was collected and filtered through a 0.22 μm mixed cellulose nitrate filter. HPLC (Thermo U3000, Thermo Fisher Scientific, Germering, Germany) equipped with a GOLD C8 column (250 × 4.6 mm, 5 μm, Thermo Scientific Hypersil) was used to separate selenoamino acids with a mobile phase (20 mM KH_2_PO_4_, 5% methanol, 0.05% heptafluorobutyric acid) at a flow rate of 1.2 mL/min, and the retention times for ^80^Se isotopes were simultaneously monitored by ICP-MS. Peaks were identified according to the retention times of the standards (SeCys_2_, MeSeCys, and SeMet). The identified selenoamino acids were quantified based on the peak areas of the standard compounds.

### 2.7. Volatile Se Compound Analysis

The supernatant (3 mL) was precisely sampled in a 20 mL headspace bottle, sealed, and placed on the headspace sampler for headspace-solid phase microextraction-gas chromatography-mass spectrometry (HS-SPME-GC-MS, TSQ8000, Thermo Fisher Scientific Inc. Austin, TX, USA) equipped with an HP-5 (60 m × 0.25 mm × 0.25 μm) column under the following conditions. The extraction head was pretreated at the GC inlet until there was no impurity peak and then extracted at 55 °C for 30 min. The adsorbed volatile compounds were desorbed at 250 °C for 5 min at the GC injector and injected into the GC column. At the same time, the instrument was used to collect data. The following parameters were used: extraction head, DVB/CAR/PMDS; carrier gas, helium, 1.2 mL/min; programmed temperature conditions, 40 °C for 1 min, 4 °C/min to 120 °C, and 10 °C/min to 240 °C for 6 min; transfer line temperature, 280 °C; ion source temperature, 300 °C; ion potential, 70 eV; ionization mode, EI+; quality scanning range *m*/*z*, 33–350 amu. Volatile Se-compounds were identified by comparing the identified mass spectra with the NIST 2017 library using the retention time index and *m*/*z* of the fragments. The relative content of each identified volatile Se compound was expressed as the relative percentile (*n* = 3) of the dimethyl selenide peak areas (day 4 in the 50 ppm group).

### 2.8. Statistical Analysis

All experiments were performed in triplicate. The results were indicated as the mean ± standard deviation, with significance at *p* < 0.05, after subjecting data to an analysis of variance using SPSS software version 20.0.

## 3. Results

### 3.1. Growth and Morphology of G. lucidum Cultured with Different Concentrations of Na_2_SeO_3_

**Figure 1:**
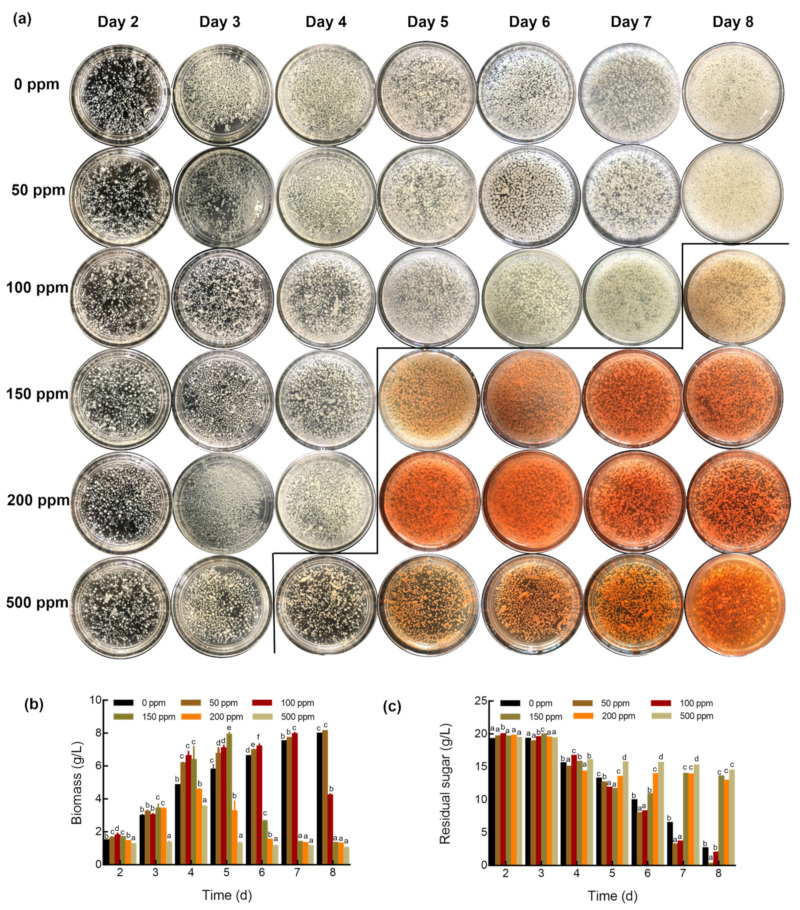
Morphology (**a**), biomass (**b**), and residual sugar (**c**) content of *Ganoderma lucidum* in the liquid culture process. The bars indicate the standard error of the mean (different letters indicate a significant difference on the same culture day at *p* < 0.05).

The influence of different concentrations of Na_2_SeO_3_ on mycelial growth was examined. Visible indicators were noted with Na_2_SeO_3_ concentrations >100 ppm, specifically with the mycelia color turning from white to bright red on days 8, 5, 5, and 4 with the addition of 100, 150, 200, and 500 ppm Na_2_SeO_3_, respectively. The red color indicated the formation of elemental Se in the mycelia. Moreover, it also gave off an unpleasant odor accompanied by the mycelial color change. No distinct differences in color, odor, and shape were observed between the mycelia cultured with 50 ppm Na_2_SeO_3_ and with no Na_2_SeO_3_ (Figure 1a).

The biomass yield of *G. lucidum* varied considerably in response to different levels of Se. As shown in Figure 1b, as compared with no Na_2_SeO_3_ addition, mycelia growth changed from being promoted to inhibited with an increase in Na_2_SeO_3_ concentration. Higher Se (IV) doses resulted in a considerable reduction in biomass compared to that with low Se content. Specifically, 50 ppm Na_2_SeO_3_ slightly promoted growth in the whole process, leading to the highest increment of 27.5% in biomass on the 5th day, whereas in the 100 ppm and 150 ppm Na_2_SeO_3_ groups, biomass initially increased, followed by a decrease, with the growth turning point occurring on the 5th and 7th days. An obvious reduction in growth was measured for *G. lucidum* in 200 ppm and 500 ppm Na_2_SeO_3_ groups, with much lower biomass, especially since the 4th day, which was also in agreement with the residual sugar consumption, with basically no consumption since the 4th day (Figure 1c). It could be clearly seen that the biomass declined at a rapid rate in the 100–500 ppm groups from the 4th to 7th day; pronounced cell damage occurred in a short time with excessive Se.

These results indicated that a low level of Na_2_SeO_3_ could be efficiently assimilated by *G. lucidum* mycelia, but there was saturation and even a suppressive role with a high Se level. Therefore, according to the growth response to selenite, 50 ppm and 200 ppm were chosen as the promoting and inhibiting Se concentrations to compare the *G. lucidum* growth and bioactive metabolites changing over the period of notable change (4th–6th day) in the liquid culture process.

### 3.2. Ultra-Structure Alterations

**Figure 2:**
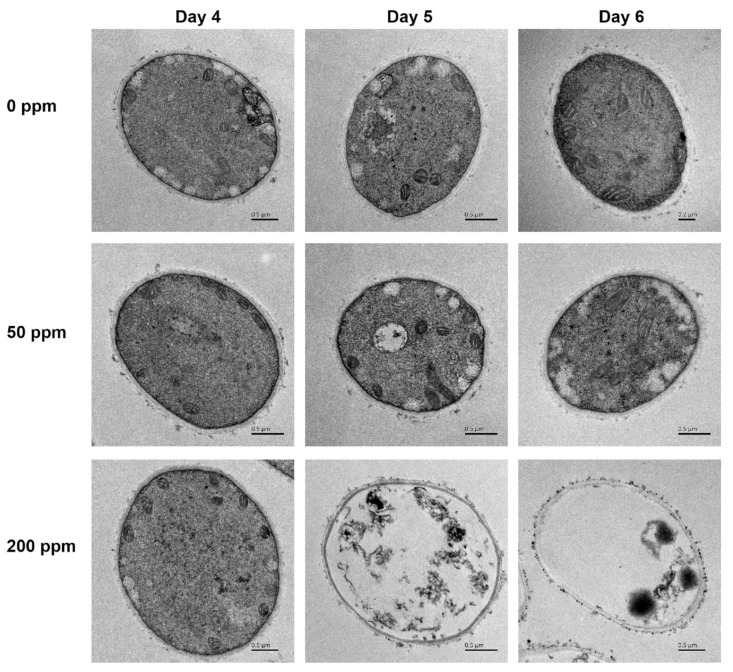
Ultra-structure of *Ganoderma lucidum* mycelia with selenite supplementation at different concentrations.

Ultra-structure analysis was performed to assess the influence of Na_2_SeO_3_ on *G. lucidum* mycelia. All organelles in mycelia cells with no Na_2_SeO_3_ addition and 50 ppm Na_2_SeO_3_ conditions were noticeably illustrious and well developed. There were clear round-shaped mitochondria and clear micrographs of the cell wall and cell membrane. No obvious variation was observed in *G. lucidum* cells treated with 200 ppm Na_2_SeO_3_ on the 4th day; noticeably, degraded cells appeared from the 5th day, the organelles inside the cell were damaged and degradation changes occurred, the electron density of the cell wall was not uniform, and cytoplasmic structure, organelles, and vacuoles disappeared; however, the cell shape was basically intact. As shown in Figure 2, small particles appeared in mycelia cells cultured with 200 ppm Na_2_SeO_3_ on the 5th and 6th days, and a clear ring of particles was noted outside the cell, which was speculated to be Se nanoparticles (SeNPs). However, no particles were observed in the cells cultured with 50 ppm Na_2_SeO_3_. It could be seen that the application of 200 ppm Na_2_SeO_3_ strongly damaged the ultrastructural profile, and the damage degree increased with the culture process, whereas there were no distinctions between the no Na_2_SeO_3_ and 50 ppm Na_2_SeO_3_ addition groups. This suggested that under the pressure of a high Se dosage, a “toxic effect” was exerted in mycelia, the organelles were seriously damaged, and insoluble substances were released from the cells, after which empty cells appeared.

### 3.3. Changes in Mycelial Cell Chemical Composition

**Figure 3:**
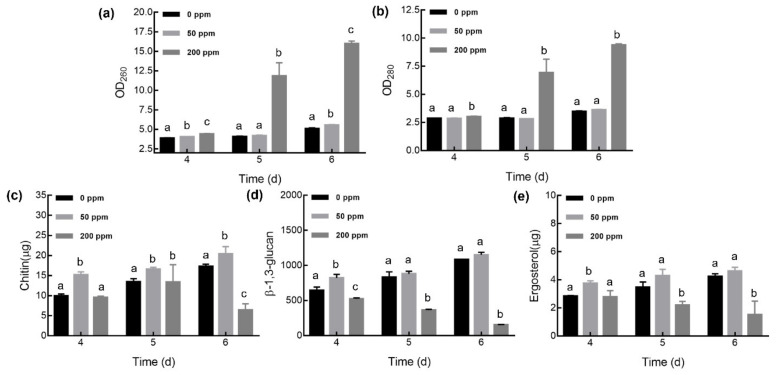
Changes in nucleic acid (**a**) and protein (**b**) and the cell wall and cell membrane components chitin (**c**), β-1,3-glucan (**d**), and ergosterol (**e**) in *Ganoderma lucidum* with selenite at different concentrations. The bars indicate the standard error of the mean (different letters indicate a significant difference on the same culture day at *p* < 0.05).

Quantitative analysis of intracellular nucleic acid and protein, cell wall components (chitin and β-1,3-glucan), and a cell membrane component (ergosterol) were performed to detect the cell structure and physiological state caused by Se supplementation (Figure 3).

For Se supplementation at 50 ppm, the nucleic acid and protein did not leak outside the cell during the culture process. In addition, chitin increased, and β-1,3-glucan and ergosterol remained unchanged. However, in mycelia cultured with 200 ppm Na_2_SeO_3_, nucleic acid and protein leakage obviously occurred since the 5th day (*p* < 0.05); compared to that with the control, the nucleic acid leakage increased by 1.9- and 2.2-folds on the 5th and 6th days, respectively, whereas protein leakage increased by 1.4- and 1.7-folds. In addition, the contents of the three components were lower than those of the control and 50 ppm Na_2_SeO_3_ groups, especially since the 5th day (*p* < 0.05). Therefore, the mycelial cell wall and cell membrane were destructed under high Se conditions, and the degree of damage potentiated with the extension of culture time. Consistent with the TEM results, the membrane integrity and permeability of *G. lucidum* mycelia cells were damaged with high Na_2_SeO_3_ concentrations, resulting in leakage of the cytoplasm (nucleic acid and protein). However, a 50 ppm Na_2_SeO_3_ level had no negative effects on the mycelia.

### 3.4. Volatile Se Compounds in G. lucidum

With the extension of culture time, the mycelia color and odor changed, especially in *G. lucidum* supplemented with 200 ppm Na_2_SeO_3_. Therefore, to fully explore the odor changes of *G. lucidum* under Na_2_SeO_3_ culture, headspace solid-phase microextraction combined with gas chromatography-mass spectrometry was used to analyze the volatile Se compounds in the liquid culture process (Supplementary Figure S1). Significant differences in the volatile Se compound profiles were observed in response to Se treatments, and the kinds and contents of volatile Se compounds in the 200 ppm Na_2_SeO_3_ group were remarkably higher than those in the 50 ppm Na_2_SeO_3_ group. Ten volatile Se compounds were identified in the culture broth of Se-enriched *G. lucidum*, and carbo selenide, dimethyl selenodisulfide, and dimethyl diselenenyl sulfide were sulfur-containing compounds only detected in the 200 ppm Na_2_SeO_3_ group. No volatile Se compounds were found in the control condition, whereas three and ten volatile Se compounds were identified in the 50 ppm and 200 ppm Na_2_SeO_3_ groups, respectively (Table 1).

The volatile Se compound types and contents all increased with increasing Na_2_SeO_3_ levels, which was not only the main cause of the odor change but also the detoxification mechanism of *G. lucidum* to alleviate high Se concentration stress. The most abundant volatile Se compound was dimethyl selenide in the 50 ppm Na_2_SeO_3_ group, accounting for more than 90% of all volatile Se compounds. Carbon diselenide, methyl diselenide, dimethyl diselenenylsulfide, and triselenothane were the major volatile Se compounds in the 200 ppm Na_2_SeO_3_ group, and carbon diselenide was principally produced on the 4th day during the culture process, whereas dimethyl diselenenylsulfide, methyl diselenide, and triselenothane were largely generated on the 5th and 6th days.

### 3.5. Bioactive Metabolites

#### 3.5.1. Polysaccharide

Polysaccharide, a principal bioactive metabolite in *G. lucidum* with excellent bioactivities, has been the research focus of mushrooms in recent years [21]. Se application affected polysaccharide metabolism as follows: 50 ppm Na_2_SeO_3_ culture increased polysaccharide production, the percentage was approximately 9–25%; 200 ppm Na_2_SeO_3_ application showed no impact on polysaccharide yield on the 4th day but a dramatic reduction in polysaccharide production was observed from the 5th day in this process (Table 2), and the decrease was recorded by 39–79% and 51–77% lower than those in the no Na_2_SeO_3_ and 50 ppm Na_2_SeO_3_ addition samples.

#### 3.5.2. Se Enrichment Capacity

The Se accumulated in mycelia increased throughout the liquid culture process, and it progressively enhanced with increasing Na_2_SeO_3_ doses added to the culture (Table 2). The total Se accumulated in *G. lucidum* supplied with 50 ppm and 200 ppm Na_2_SeO_3_ reached the maximum values of 421.69 and 41,574.27 mg/kg on the 6th day, respectively, demonstrating that a higher Se treatment could improve Se accumulation ability and that *G. lucidum* was a good Se carrier.

#### 3.5.3. Selenoamino Acids

HPLC-ICP-MS was used to detect the selenoamino acids in *G. lucidum* and the chromatograms were shown in Supplementary Figure S2. Three peaks with the same retention times as those observed for SeCys_2_, MeSeCys, and SeMet standards were obtained in *G. lucidum* cultured with 50 ppm and 200 ppm Na_2_SeO_3_. As two main Se-conjugated amino acids, a higher proportion of SeMet than SeCys_2_ was measured in the enzyme-hydrolyzed *G. lucidum* mycelia extract.

The contents of SeCys_2_, SeMet, and MeSeCys all increased in the liquid culture process in *G. lucidum* supplemented with 50 ppm Na_2_SeO_3_, achieving the maximum values of 92.01, 445.83, and 54.44 mg/kg, respectively. In *G. lucidum* supplied with 200 ppm Na_2_SeO_3_, the SeCys_2_ and SeMet content reached a maximum of 237.84 and 301.78 mg/kg, respectively, on the 4th day during the culture process, which was followed by a decrease, MeSeCys showed a dramatic increase, being the highest on the 6th day with 304.04 mg/kg. The MeSeCys content was five folds higher than that of *G. lucidum* with 50 ppm Na_2_SeO_3_. The Se proportion of the three selenoamino acids accounted for 55.72% of the total Se content in the 50 ppm Na_2_SeO_3_ group, but the proportion was only 19.21% on the 4th day and then decreased greatly in the 200 ppm Na_2_SeO_3_ group (Table 2).

Consequently, a low Se dose was optimal for selenoamino acid production during the entire process, and SeMet was the major component for *G. lucidum*. However, with a high Se content, the selenoamino acid proportions were profoundly changed. SeMet, and SeCys_2_ decreased, while as the product of SeCys methylation, MeSeCys increased in this process and was the main component on the 6th day. Hence, *G. lucidum* was able to take up Na_2_SeO_3_ directly and transformed it into organic Se.

## 4. Discussion

Edible and medicinal mushrooms are good Se accumulators with high nutritional value and biological activity, and Se biofortification with mushrooms is an efficient strategy to enhance human Se intake. It is a promising application to obtain large mycelia yields or bioactive metabolites, via liquid culture technology, and then to develop them into various functional foods. Many studies have shown that Se can significantly affect mushroom growth and mycelium development [12,22]. This study found that an appropriate amount of Se was favorable for *G. lucidum* growth. During the liquid culture process, 50 ppm Na_2_SeO_3_ was tested as the optimal concentration for growth, and it did not cause morphological changes in *G. lucidum*, but Na_2_SeO_3_ at concentrations >100 ppm negatively affected mycelial growth. To satisfy human dietary Se requirements, the *G. lucidum* mycelia cultured with 50 ppm selenite could be used as dietary supplements. According to the recommended daily intake of Se 60 μg for adults [23], consuming 140 mg *G. lucidum* mycelium per day will maintain human health and prevent diseases related to Se deficiency, there including 62.42 μg SeMet and 12.88 μg SeCys_2_.

Mycelium morphology and productivity are highly interlinked, and these associations have been investigated [24]. The *G. lucidum* mycelia cell ultrastructures were damaged with 200 ppm Na_2_SeO_3_ since the 5th day, including the cell wall, cell membrane, and organelles. Carbon and nitrogen metabolism were seriously disturbed, further influencing the growth and bioactive metabolites, whereas 50 ppm Na_2_SeO_3_ did not cause obvious morphological changes. Mushrooms can grow over a wide concentration range of Se, and their tolerance capacity to Se alters with mushroom species, culture conditions, Se sources, and doses. *Hericium erinaceum* mycelia were also found to be degraded when cultivated with 100 ppm Na_2_SeO_3_, disturbing the normal respiratory physiological process in cells. Further, 20 g/kg Selol was determined to contribute to the thickening of the cell wall, which implied an influence on polysaccharide production [25]. Thus, changes in mycelium morphology can be a direct indicator of Se stress to edible and medicinal mushrooms.

Volatile substances are essential for the flavor of edible and medicinal mushrooms [26]. Investigations into the odor compositions are crucial for mushrooms, and volatile Se compounds are often neglected because they can easily be lost during sample analyses. Biomethylation is a bioprocess that plays an important role in mushroom Se metabolism and produces volatile Se compounds such as dimethyl selenide (DMSe) and dimethyl diselenide (DMDSe), which will lead to odor change. With the increase in Na_2_SeO_3_ concentration, the types and contents of volatile Se compounds released by *G. lucidum* in the liquid culture increased distinctly, ultimately resulting in a strong stimulating odor. As many as ten volatile Se compounds have been detected in *G. lucidum*, including dimethyl selenide and methyl diselenide, which were the main volatile Se metabolites produced by plants as a result of the phyto-volatilization or biomethylation of Se [27]. Volatile organic sulfur and Se compounds detected in bottled drinking water were reported to have swampy, rotten egg, sulfidic, cooked vegetable, and/or cabbage odors [28]. The production of volatile Se compounds is a self-detoxification mechanism that occurs under high Se conditions, and the concentration and toxicity of inorganic Se compounds can be decreased by the production of volatile Se compounds [29]. Understanding the profile of volatile Se compounds would contribute to disclosing the physiological status of mushrooms during the Se enrichment process, which will help to shed light on the mechanisms of Se toxicity and have implications for the Se biofortification. It remains to be determined whether these volatile Se compounds are common metabolites in other mushroom species cultured with Se.

As one of the most important bioactive metabolites of *G. lucidum*, polysaccharide synthesis was greatly influenced by Na_2_SeO_3_ addition, especially under high Se dose conditions. With the increase in Na_2_SeO_3_ concentrations, polysaccharide production was increased followed by a decrease. Specifically, 50 ppm Na_2_SeO_3_ promoted polysaccharide synthesis, but 200 ppm Na_2_SeO_3_ significantly inhibited its synthesis after the 5th day, which was associated with cell degradation. This result was consistent with findings that 10 μg/mL Na_2_SeO_3_ promoted the production of *Lentinula edodes* mycelium exopolysaccharide (EPS), whereas 20 μg/mL Na_2_SeO_3_ reduced EPS [30]. The addition of 7 mg/L Na_2_SeO_3_ enhanced polysaccharide biosynthesis in *Cordyceps militaris*, and the key enzymes PGM, PGI, and UGP involved in polysaccharide synthesis were found with elevated gene expression in the Se-enriched group [31]. Presently, there have been numerous studies aiming to improve mushroom polysaccharide yield, with a 62.50% increment in *G. lucidum* after the addition of L-phenylalanine [17]. Hence, low concentrations of Se addition in the liquid culture is a viable strategy, which not only positively affects the polysaccharide yield but also increases the active substances of organic Se.

Organic Se is more bioavailable or bioactive than inorganic Se in humans. SeMet is more bioaccessible than most other organic Se compounds such as SeCys_2_ [32]. The Se sources and doses supplied in the media affected the selenoamino acid composition and proportion. In addition, different mushrooms have diverse selenoamino acid types. SeMet and MeSeCys reached their maximum values when irrigated with 20 mg Se/L, whereas 10 mg Se/L was required for SeCys in *Agaricus bisporus* fruiting bodies [33]. Studies have also shown that mushrooms such as *Pleurotus ostreatus* [34] and *L. edodes* [35], given Se fortification, yield good bioaccessibility in the form of SeMet. Moreover, MeSeCys was identified in *H. erinaceus* [12], *Auricularia auricular* [7], and *P. ostreatus* [34] fruiting bodies. Here, 50 ppm Na_2_SeO_3_ was beneficial for SeCys_2_ and SeMet production throughout the whole process, whereas the content of SeCys_2_ and SeMet decreased in *G. lucidum* cultured with 200 ppm Na_2_SeO_3_. Tolerance to high Se in Se-hyperaccumulating plants is correlated with the ability to biosynthesize MeSeCys, an amino acid derivative that is not incorporated into proteins [36]. The same phenomenon was observed for *G. lucidum*; MeSeCys content increased with the addition of 200 ppm Na_2_SeO_3_, which was produced to alleviate the negative effects.

*G. lucidum* mycelia were bright red, especially under high Se conditions, and it was speculated that they could convert Na_2_SeO_3_ into SeNPs. Similarly, *L. edodes* has also been reported to transform Se into SeNPs, and SeNPs are localized inside the mycelial hyphae [37]. SeNPs have been used in the maintenance of human health [38]. They can not only be absorbed and used by humans but also have unique functions, such as antioxidant, immune-regulation, anti-cancer, and antibacterial activities. SeNPs produced by microorganisms are often characterized by low toxicity and good stability, and they form Se^0^ aggregates alone or in combination with exopolysaccharides and proteins [39,40]. The transition of mycelia color from white to red was the starting point of notable changes in cells and metabolites, which was also the most intuitive embodiment of complex changes in cells.

Mycelial growth and morphology can serve as indicators of Se stress in mushrooms. The incorporation of Se into the mycelia caused variations in morphology and growth in a manner dependent on the Se dose. To obtain different Se-enriched *G. lucidum,* the liquid culture can be controlled by the color changing (time) under the pressure of high Se concentrations. Se-enriched *G. lucidum* mycelia obtained in liquid culture could possibly be used as a source of Se for human consumption. To obtain more high-value Se-enriched *G. lucidum*, further studies should explore ways to improve the Se enrichment capacity, enhance bioactive metabolite production, and reduce the toxic effect, carefully evaluate the applied Se biofortification strategies and cost-effective parameters, and balance the relationship between them.

## 5. Conclusions

*G. lucidum* is an excellent Se carrier, not only rich in natural bioactive metabolites, but also could transform inorganic Se into organic Se with the advantage of efficacy. The growth and bioactive metabolites of *G. lucidum* were greatly altered by the application of Na_2_SeO_3_ in the liquid culture process. Specifically, 50 ppm Na_2_SeO_3_ did not influence growth, but 200 ppm Na_2_SeO_3_ could impose toxic effects on mycelia growth and bioactive metabolites, especially on the 4th–6th days of mycelia development. (i) Mycelia growth and ultrastructure, odor, and color were basically unchanged with 50 ppm Na_2_SeO_3_, but decreased biomass, degraded mycelial cells, a red color, and an unpleasant odor were the main characteristics of *G. lucidum* with 200 ppm Na_2_SeO_3_. (ii) Ten volatile Se compounds were identified in the culture broth of Se-enriched *G. lucidum,* which led to an odor change. (iii) The total Se accumulated in *G. lucidum* was enhanced with the increased levels of Na_2_SeO_3_, and SeMet was the major selenoamino acid in *G. lucidum* with 50 ppm Na_2_SeO_3_, but SeMet and SeCys_2_ content gradually declined and MeSeCys increased in this process in the 200 ppm Na_2_SeO_3_ group. Further, polysaccharide yields were promoted and inhibited with 50 and 200 ppm Na_2_SeO_3_, respectively. This study provides further insights into our understanding of the growth and bioactive metabolite alternations of *G. lucidum* caused by Se addition in the liquid culture process and is useful for the production of functional Se-enriched mushrooms.

## Figures and Tables

**Table 1:** Volatile Se compounds of *Ganoderma lucidum* in the liquid culture process.

Se Compound	50 ppm		
	Day 4	Day 5	Day 6
Dimethyl selenide	1.000 ^b^	1.748 ± 0.027 ^b^	1.634 ± 0.046 ^b^
Carbo selenide	ND	ND	0.088 ± 0.006 ^a^
Dimethyl selenone	0.053 ± 0.001 ^a^	0.115 ± 0.004 ^a^	0.170 ± 0.009 ^a^
**Se Compound**	**200 ppm**		
	**Day 4**	**Day 5**	**Day 6**
Dimethyl selenide	0.689 ± 0.097 ^a^	0.596 ± 0.007 ^a^	0.134 ± 0.017 ^a^
Carbo selenide	0.765 ± 0.027 ^a^	0.681 ± 0.068 ^a^	0.245 ± 0.002 ^b^
Carbon diselenide	9.467 ± 0.012	0.669 ± 0.071	0.313 ± 0.082
Methylselenoacetate	ND	0.235 ± 0.005	0.052 ± 0.012
Dimethyl selenone	0.132 ± 0.009 ^b^	4.092 ± 0.015 ^b^	4.990 ± 0.601 ^b^
Methyl diselenide	0.538 ± 0.069	35.329 ± 0.032	44.242 ± 0.022
1-(methylselanyl)propan-2-one	0.068 ± 0.008	0.108 ± 0.011	0.080 ± 0.015
Dimethyl selenodisulfide	ND	ND	0.058 ± 0.006
Dimethyl diselenenyl_sulfide	0.081 ± 0.002	6.925 ± 0.013	6.813 ± 0.068
Triselenothane	ND	21.495 ± 0.053	31.129 ± 0.021

ND: not detected. Results are expressed as the relative percentile (*n* = 3) of the dimethyl selenide peak areas (day 4 in the 50 ppm group). Different letters indicate a significant difference for the same compound in different selenite-treated groups on the same culture day at *p* < 0.05.

**Table 2:** Polysaccharide, total Se content, and selenoamino acids in *Ganoderma lucidum.*

Bioactive Metabolites		Day 4	Day 5	Day 6
Polysaccharide (mg/L)	0 ppm	269.81 ± 16.81 ^a^	560.12 ± 14.67 ^b^	1253.42 ± 9.72 ^b^
	50 ppm	331.21 ± 3.89 ^b^	702.81 ± 25.68 ^c^	1365.97 ± 10.93 ^c^
	200 ppm	262.23 ± 8.06 ^a^	340.93 ± 28.81 ^a^	259.11 ± 32.86 ^a^
Se_(total)_ (mg/kg)	0 ppm	2.92 ± 0.65 ^a^	3.31 ± 0.89 ^a^	2.86 ± 0.23 ^a^
	50 ppm	275.11 ± 27.39 ^b^	316.87 ± 30.45 ^a^	421.69 ± 29.07 ^a^
	200 ppm	1398.41 ± 126.96^c^	22,472.65 ± 1271.83 ^b^	41,574.27 ± 1933.50 ^b^
SeCys_2_ (mg/kg)	50 ppm	55.37 ± 0.39 ^a^	82.85 ± 4.52 ^a^	92.01 ± 3.22 ^a^
SeMet (mg/kg)		146.55 ± 7.47 ^a^	243.96 ± 9.31 ^a^	445.83 ± 30.95 ^a^
MeSeCys (mg/kg)		39.99 ± 0.42 ^a^	40.69 ± 0.57 ^a^	54.44 ± 1.25 ^a^
SeCys_2_ (mg/kg)	200 ppm	237.84 ± 13.22 ^b^	177.23 ± 12.21 ^b^	160.99 ± 14.51 ^b^
SeMet (mg/kg)		301.78. ± 10.41 ^b^	157.25 ± 8.66 ^b^	149.44 ± 29.16 ^b^
MeSeCys (mg/kg)		72.23 ± 5.07 ^b^	104.00 ± 5.11 ^b^	304.04 ± 30.71 ^b^

Different letters indicate a significant difference of the same compound in different selenite-treated groups on the same culture day at *p* < 0.05.

## Supplementary Materials

The following are available online at https://www.mdpi.com/article/10.3390/foods10081860/s1, Figure S1: GC-MS total ion map and structures of the identified volatile Se-compounds, Figure S2: HPLC-ICP-MS map of the selenoamino acids. (a) standards; (b–d) *G. lucidum* cultured with 50 ppm Na_2_SeO_3_ from the 4th to 6th day; (e–g) *G. lucidum* cultured with 200 ppm Na_2_SeO_3_ from the 4th to 6th day.
