# Supplementary material for: Influence of Selenium Biofortification on the Growth and Bioactive Metabolites of Ganoderma lucidum
Source: Foods. 2021 Aug 12;10(8):1860. doi: 10.3390/foods10081860 (PMC8391904; doi:10.3390/foods10081860)
Supplement: Supplementary file 1 [file foods-10-01860-s001.zip › foods-1296445-supplementary.pdf]

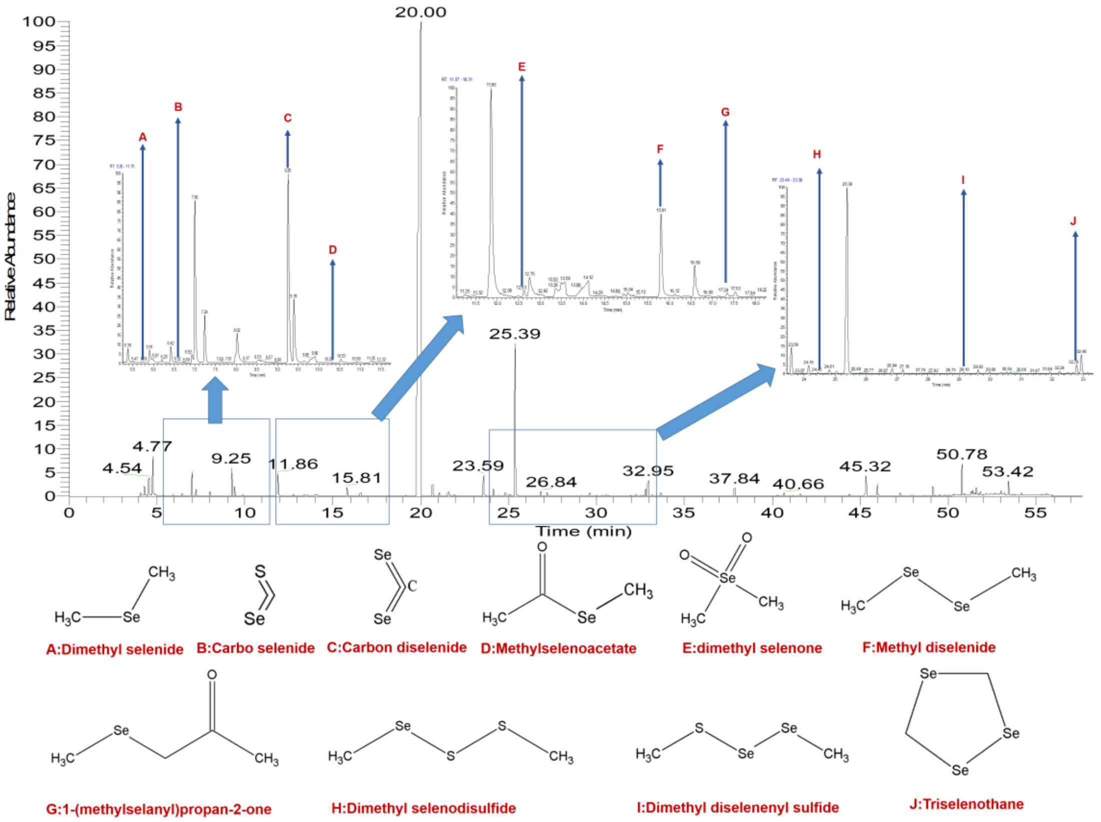

Fig. S1 GC-MS total ion map and structures of the identified volatile Se-compounds

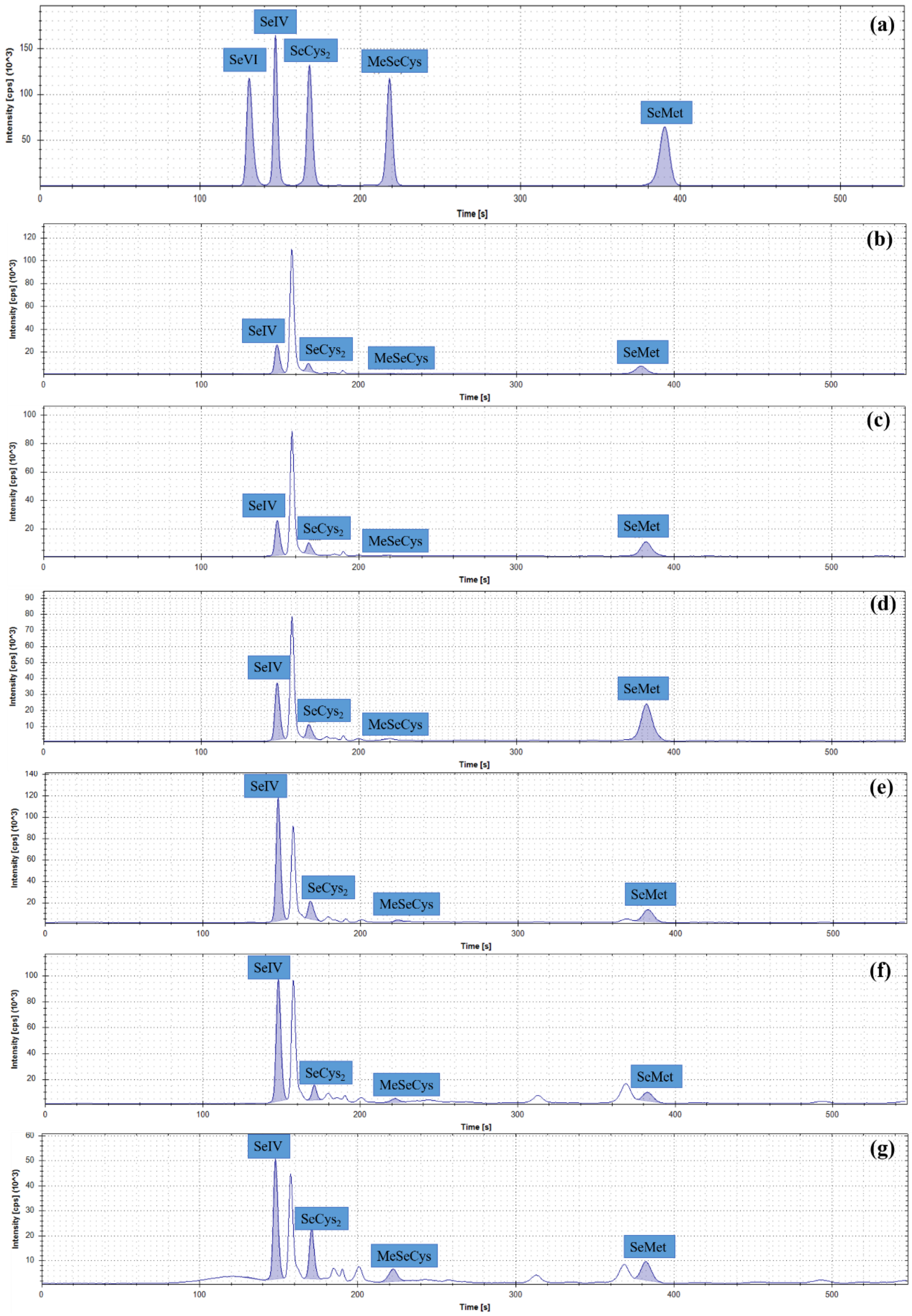

Fig. S2 HPLC-ICP-MS map of the selenoamino acids. (a) standards; (b-d) *G. lucidum* cultured with 50 ppm Na_2SeO_3 from the 4th to 6th day; (e-g) *G. lucidum* cultured with 200 ppm Na_2SeO_3 from the 4th to 6th day.
